# Comparative Safety, Immunogenicity, and Efficacy of CEF Cell-Based and DF-1 Cell Line Adapted Infectious Bursal Disease Vaccines in Specific-Pathogen-Free Chickens

**DOI:** 10.1155/2022/5392033

**Published:** 2022-10-15

**Authors:** Daniel Workineh, Molalegne Bitew, Daniel Oluwayelu, Belayneh Getachew, Takele Abayneh, Esayas Gelaye, Hawa Mohammed, Tedros Fikru, Mastewal Birhan, Bereket Dessalegn, Getaw Deresse, Kassaye Adamu, Saddam Mohammed Ibrahim

**Affiliations:** ^1^Pan-African University for Life and Earth Sciences Institute, Ibadan, Nigeria; ^2^Veterinary Teaching Hospital, College of Veterinary Medicine and Animal Sciences, University of Gondar, Gondar, Ethiopia; ^3^Bio and Emerging Technology Institute, Addis Ababa, Ethiopia; ^4^Department of Veterinary Microbiology, University of Ibadan, Ibadan, Nigeria; ^5^National Veterinary Institute, Bishoftu, Ethiopia; ^6^Department of Veterinary Pathobiology, College of Veterinary Medicine and Animal Sciences, University of Gondar, Gondar, Ethiopia

## Abstract

Infectious bursal disease (IBD) is an immunosuppressive and economically important disease of young chickens caused by infectious bursal disease virus (IBDV). The National Veterinary Institute (Bishoftu, Ethiopia) produces intermediate IBDV vaccine using primary chicken embryo fibroblast (CEF) cells, a method with technical and economical cumbersome. This study assessed the safety, immunogenicity, and efficacy of DF-1 cell line-adapted IBDV LC–75 vaccine strain in reference to the CEF-based vaccine. Confluent monolayer of DF-1 cells was infected with IBDV and cells with cytopathic effects were passaged until 3^rd^ passage. Viral growth was confirmed using a one-step RT-PCR targeting IBDV VP2 gene. Viral titer increased from 1^st^ passage through 3^rd^ passage. Safety was assessed in 30 specific-pathogen-free chickens (15 chickens/group) injected with 10-fold field dose of each vaccine intraocularly and monitored for 21 days. For immunogenicity and efficacy, 60 specific-pathogen-free chickens were grouped into 3 (20 chickens/group). First and 2^nd^ group received DF-1 cell and CEF-based IBDV vaccines, respectively. The 3^rd^ group served as unvaccinated control. Antibody response was measured using iELISA. Chickens were challenged 4 weeks postvaccination with very virulent IBDV (vvIBDV) intraocularly and followed-up for 10 days. Vaccination did not cause any adverse reactions during the 21 days of follow-up. In addition, both vaccines induced higher antibody titer 14 and 24 days-post-vaccination as compared to unvaccinated controls (*p* < 0.05). Moreover, DF-1 and CEF-based IBDV LC–75 vaccines rendered a complete protection against vvIBDV. Contrarily, morbidity and mortality in unvaccinated chickens was 50% and 30%, respectively. The results indicated that DF-1 and CEF cell-based IBDV vaccines are comparably immunogenic and efficacious. Therefore, DF-1 cell-line can be considered an affordable and convenient alternative to the CEF-based approach. The suitability of DF-1 cells to grow other IBDV strains and safety of these vaccines on bursa of Fabricius should further be investigated.

## 1. Introduction

Infectious bursal disease (IBD) a.k.a. Gumboro disease, is a highly contagious and immunosuppressive viral infection of young chickens with marked economic impact on poultry production [1–4]. IBD is caused by infectious bursal disease virus (IBDV), a highly mutant, environmentally resistant, nonenveloped, and bisegmented (segment A and B) double stranded RNA (dsRNA) virus belonging to the genus *Avibirinavirus* and the family *Birnaviridae* [5, 6]. The virus is known to have two serotypes, serotype 1 [1] and serotype 2 [7, 8], with only serotype 1 capable of causing disease in chickens.

Traditionally, isolates of serotype 1 are categorized into subclinical (sc), classical virulent (cv), and very virulent (vv) IBDV, depending on their severity [9]. However, such classification based on antigenic types and pathotypes has been ambiguous. For example, several subtypes have been formed within the two major antigenic groups (classical and variant) as a result of antigenic drift [10]. In addition, pathotype wise, several natural reassortants of vvIBDV, and serotype 1 non-vvIBDV [11, 12] and serotype 2 [13] with less mortality or virulence profile than the typical vvIBDV have been reported globally.

Consequently, IBDV has recently been classified into seven different genogroups (G1-G7) based on sequences of the hypervariable region of the capsid protein, VP2 (hvVP2) [14]. IBDV primarily replicates in developing B-cells within bursa of Fabricius (BF) resulting in massive lymphocytic depletion [15, 16], thus, humoral immunosuppression, which renders chickens vulnerable to opportunistic pathogens and impairing response to vaccination [17, 18]. It has been reported that IBDV-infected flocks had higher mortality, bursal atrophy, poorer feed conversion ratio (FCR), and decreased meat production [19]. Therefore, IBD is a disease of severe economic consequence.

Due to the resistant nature of the virus, sanitary measures commonly applied on poultry farms are not sufficient to prevent the infection [20]. For this reason, vaccination is the key component of IBD control and prevention strategies. Currently, conventional (live-attenuated and killed-whole virus), IBDV immune complex, and viral vectored IBD vaccines are available on market. Several other platforms such as subunit, DNA, and genetically engineered IBD live vaccines have been tested with various success and are reviewed elsewhere [21]. Live attenuated IBD vaccines can be classified as mild, intermediate, intermediate plus, and hot, depending on their degree of attenuation [22]. Mild IBD vaccines are safe, but less efficacious in the presence of maternally derived antibodies (MDAs) and against vvIBDV. Intermediate, intermediate plus, hot IBD vaccines resist interference by MDAs, thus, have better efficacy. However, these vaccines cause a varying degree of bursal atrophy and immunosuppression [21, 23].

IBDV is an important poultry pathogen affecting poultry production all over Ethiopia [24–30]. Control of the disease in the country is largely reliant on vaccination using locally produced (National Veterinary Institute (NVI), Bishoftu, Ethiopia) intermediate plus IBDV vaccine derived from LC–75 strain adapted in chicken embryo fibroblast (CEF) cell sourced from specific pathogen free (SPF) eggs [28]. However, CEF-based method of production requires SPF eggs and preparation of primary cell cultures (with finite in vitro life span) which is labor intensive, expensive, and time consuming with a low viral yield making them unsuitable for continuous demand as in case of vaccine production [31]. This necessitates the industry to switch into a more affordable and convenient technique of production. Accordingly, Kebede et al. [32] has reported a Vero cell-adapted IBDV vaccine as a manageable and reproducible substitute to the current CEF cell-based IBDV vaccine. The use of cell types originating from target or closely related hosts (e.g., DF-1 cell line for avian pathogens) could be a better substitute of Vero cells for propagation of IBDV as it overcomes virus adaptation problem, thus, could facilitates better vaccine production at the industrial scale. In this study, we evaluated the safety, immunogenicity, and efficacy of DF-1 cell line adapted IBDV LC–75 vaccine strain in SPF white leghorns in comparison with the locally produced CEF-based IBDV vaccine, as an affordable, convenient, and reliable alternative IBDV vaccine production method.

## 2. Materials and Methods

### 2.1. Cells, Vaccine Strain, and Challenge Virus Strain

The DF-1 cells were grown in 75 cm^2^ tissue culture flasks with Dulbecco's Modified Eagle's Medium (DMEM) (HyClone, USA) supplemented with 10% fetal calf serum (FCS) (Gibco) and 1% antibiotics (penicillin and streptomycin). The cells were passaged using Dulbecco's phosphate buffered saline (D-PBS), 0.25% trypsin (1X), and DMEM, and incubated at 37°C in an atmosphere of 5% CO_2_ [31]. The DF-1 is spontaneously immortalized cell line derived from East Lansing Line 0 (ev-0) leghorn layer embryos (Avian Disease and Oncology Laboratory, East Lansing, MI) and is free of endogenous sequences related to the Avian Sarcoma and Leukosis Virus (ALSV) group [33, 34]. The IBDV intermediate plus LC–75 vaccine strain (genogroup 1 (A1B1), accession number: EF429252) obtained from the vaccine quality control department of the NVI, was adapted to DF-1 (Douglas Foster-1) cell line. The field isolate vvIBDV (accession number: JF826453) was propagated using chicken fibroblast cell originated from 11-day-old SPF embryonated eggs and was used as a challenge strain at a titer of 10^5.4^ 50% tissue culture infectivity dose (TCID_50_/ml) to assess vaccine efficacy. CEF cell-derived IBDV LC–75 vaccine produced by the NVI (batch number: NVI-Gum 01/20) was used a reference or comparator vaccine.

### 2.2. Adaptation of IBDV LC–75 on DF-1 Cell Line

The IBDV vaccine strain (LC-75) was reconstituted with 3 ml of DMEM, and 100 *μ*l of the suspension was inoculated at multiplicity of infection (MOI) of 0.1 into two cell culture flasks (75 cm^2^) containing confluent monolayers of DF-1 cells. One uninoculated flask served as a negative control. Flasks were incubated at 37°C in 5% CO_2_ for 7 days, and monitored twice a day for characteristic cytopathic effects (CPEs) under inverted microscope. Once CPEs were evident, the viral suspension was harvested and kept at −20°C until the next passage (Passage 1). Next day, these cells were subjected to 3 freeze-thaw cycles, and 100 *μ*l of the viral suspension was inoculated onto a confluent monolayer of DF-1 cells (two 75 cm^2^ cell culture flasks) while uninoculated flask served as a negative control. Flasks were then incubated at 37°C in 5% CO_2_ for 7 days. Then, the viral suspensions were harvested and kept at −20°C for 24 hours (Passage 2). Same procedure was followed to produce the third passage. The CPEs observed at day 5 postinoculation were highly prominent and almost 80% of the cells were infected by the IBDV vaccine strain. An infected monolayer (1 ml) from each passage was removed from the flask and transferred to Eppendorf tubes for further processing. The culture medium was centrifuged at 1800 × g for 10 min at 4°C to pellet cell debris. The clear supernatant was collected carefully, divided into aliquots, and stored at 4°C as viral stock for further use [31] (RT-PCR and titration).

### 2.3. Titration of IBDV LC–75

For viral titration, a ten-fold serial dilution (10^−1^ to 10^−9^) of each passage (1, 2, and 3) was performed by mixing 0.5 ml viral suspension with 4.5 ml of DMEM. Then, 100 *μ*l of each viral dilution was dispensed into 96 well microplate (Thermo Scientific™ Nunc) containing 100 *μ*l of DF-1 cells per well with 9 replicates for each dilution (columns 1-9). Column 10 was left empty, while columns 11 and 12 were inoculated with only cells and served as negative controls. The plate was then sealed with a microplate sealer and incubated at 37°C in 5% CO_2_. The inoculated plates were monitored under inverted microscope daily for eight days beginning from 72 h postinoculation. The viral titer at each virus passage was determined using the Reed and Muench method to determine TCID_50_ [35].

### 2.4. Molecular Confirmation of DF-1 Cell Line Adapted and CEF-Based IBDV

#### 2.4.1. RNA Extraction and Reverse Transcription Polymerase Chain Reaction (RT-PCR)

Viral RNA of the vaccine strains propagated on both DF-1 and CEF cells was extracted using the Qiagen RNeasy® Mini Kit (cat. nos. 74104 and 74106) based on the supplier's instructions. The master mix comprised eight reactions including a negative control, a positive control, two extraction controls, and four samples.

A one-step RT-PCR was carried out according to manufacturer's instruction by amplifying a 400 bp of the hypervariable region (HVR) of the IBDV VP2 gene. Primers used were IBD VP2 PANVAC-4, F: 5′-TCTTGGGTATGTGAGGCTTG-3′ and IBD VP2 PANVAC-5, R: 5′-CCCGGATTATGTCTTTGA-3′. A master mix with a final volume of 25 *μ*l was prepared in a 0.2 ml capacity thin-well PCR tubes with the following reagents: 4 *μ*l of RNase free water, 5 *μ*l of Q-solution (5×), 5 *μ*l of PCR buffer (5×), 2 *μ*l each of forward and reverse primer, 1 *μ*l dNTPs (10 mM), 1 *μ*l of one-step RT-PCR enzyme, and 5 *μ*l of template RNA. The PCR cycling parameters included one cycle of cDNA synthesis for 30 minutes at 55°C, one cycle of initial denaturation for 15 minutes at 95°C, 35 cycles of denaturation for 30 seconds at 95°C, annealing for 30 seconds at 55°C, elongation for 30 seconds at 72°C, and followed by one cycle of final elongation at 72°C for 7 minutes. The PCR products were visualized by 1.5% (w/v) agarose gel electrophoresis stained with gel red.

### 2.5. Experimental Chickens and Study Design

A total of 90, 14-day-old SPF, white leghorns of both sexes were used. Sixty (60) chickens randomly assigned into 3 groups (20 chickens/group) were used in the comparative immunogenicity and efficacy study, while 30 chickens divided into two groups (15 chickens/group) were used in the safety assessment. All the experiments were performed in accordance with the NVI Animal Handling and Research Ethics Guideline. Commercial feed and water were provided ad libitum.

### 2.6. Comparative Evaluation of DF-Cell Line-Adapted and CEF Cell-Based IBDV Vaccines

#### 2.6.1. Assessment of Safety

As mentioned above, 30 chickens randomly allocated to two groups of 15 chickens were used to assess the safety of the DF-1 cell line-adapted and CEF-based IBDV vaccines. Chickens in group 1 (*n* = 15) were vaccinated with 0.4 ml of DF-1 cell-based IBDV vaccine, whereas group 2 chickens received 0.4 ml of the CEF-based vaccine intraocularly (0.2 ml into each eye) using a 1 ml tuberculin syringe. The dose of the vaccines used was ten times of the regular field dose. Vaccinated chickens were then monitored for 21 days for signs of vaccination associated morbidity and mortality [36].

#### 2.6.2. Assessment of Immunogenicity and Efficacy

Sixty chickens were randomly allocated to 3 groups of 20 chickens, Group-I, -II, and -III. Chickens in Group-I were vaccinated using 0.2 ml of DF-1 cell-based IBDV vaccine (at a titer of 10^6^ TCID_50_/ml), while chickens in Group-II were vaccinated with 0.2 ml of CEF-based IBDV vaccine (NVI-Gum 01/20) (at a titer of 10^7^ TCID_50_/ml) according to OIE [36]. Both vaccines were administered via eye drop (0.1 ml into each eye) and at the age of 14-day-old. Chickens in Group-III received PBS (0.2 ml, intraocularly) and served as unvaccinated controls. Blood samples were drawn from all chickens before vaccination, and 7, 14, and 24 days postvaccination (dpv) to evaluate antibody response against each vaccine using a commercial indirect ELISA (iELISA) kit (IDvet, Grabels, France). Absorbance was determined using an ELISA microplate reader (Thermo Fisher Scientific Multiskan MCC) at 450 nm wavelength. *S*/*P* values were calculated using the formula provided by the manufacturer as

(1) $$\begin{array}{c} S/P=\frac{\text{ODS}-\text{ODNC}}{\text{ODPC}-\text{ODNC}}, \end{array}$$

where *S*/*P* is the sample to positive ratio, ODS is the optical density of sample or test sera, ODNC is the optical density of negative control serum, and ODPC is the optical density of positive control serum.

*S*/*P* values of > 0.3 (titer of > 875) and ≤ 0.3 (titer of ≤ 875) were interpreted as positive and negative, respectively, as per the manufacturer's guide.

To assess for the vaccines' efficacy, all chickens (vaccinated and nonvaccinated) were challenged at day 26 postvaccination, with locally isolated vvIBDV (JF826453) with a titer of 10^5.6^ TCID_50_/ml intraocularly (0.2 ml/chicken, 0.1 ml into each eye) based on the OIE's guide [37]. Finally, all chickens were monitored for 10 days for the presence of any morbidity or mortality related to IBDV.

### 2.7. Ethics Statement

The chick experiment was conducted at the experimental facility within the NVI. Therefore, ethical approval has been obtained from the research ethical committee of the NVI with reference number: 03/08/4/1.

### 2.8. Statistical Analysis

Data were entered into Microsoft Excel spreadsheet and analysed using SPSS version 20 (IBM Corp., Armonk, NY). One-way ANOVA was used to compare mean antibody titers of each group varying in vaccine administered and at various intervals. Differences between groups were considered statistically significant at *p* values of less than 0.05. Tukey's HSD test was applied to determine statistical difference between two groups. HSD was computed using the formula

(2) $$\begin{array}{c} \text{HSD}=q*\sqrt{\frac{\text{MSW}}{\text{nk}},} \end{array}$$

where HSD is the honestly significant difference, *q* is the studentized range distribution at *α* level of 0.05, MSW is the mean square within group, and nk is the number of subjects in each group. Differences between means of two groups that are greater than the HSD value (computed for days 0, 7, 14, and 24) were considered honestly significant difference. Kaplan-Meier survival analysis and log-rank test were computed to compare the survival of challenged chickens in vaccinated and unvaccinated groups.

## 3. Results

### 3.1. Adaptation of IBDV LC–75 on DF-1 Cell Line

**Figure 1:**
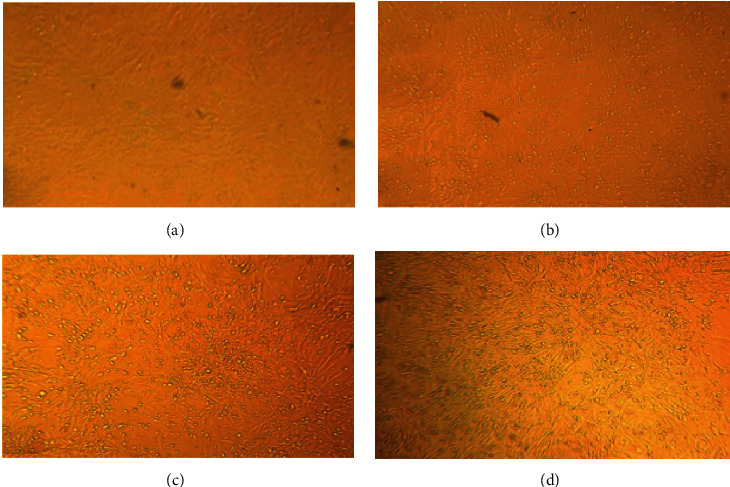
Growth of IBDV LC–75 on DF-cell lines and subsequent CPEs such as cytoplasmic granulation, cell rounding, aggregation, detachment, and floating at different passages. (a) Uninoculated DF-1 cells, (b) CPEs, passage one–day 7, (c) CPEs, passage two–day 6, and (d) CPEs, passage three–day 5.

In this study, IBDV LC–75 vaccine strain was successfully adapted on DF-1 cell line. CPEs were observed starting from 7 days postinoculation (d.p.i) at the 1^st^ passage (Figure 1(b)). CPEs continued along the 2^nd^ passage starting from 6 d.p.i (Figure 1(c)). At the 3^rd^ passage, the CPEs were evident from 5 d.p.i, and were highly prominent and characterized by cytoplasmic granulation, cell rounding, aggregation, detachment, and floating as detected under inverted microscope (Figure 1(d)).

### 3.2. Titration of IBDV LC–75 Vaccine Strain Adapted on DF-1 Cell Line

The titer of DF-1 cell line-adapted IBDV vaccine strain from each passage, i.e., passage 1, 2, and 3, was determined based on Reed and Muench's [35] technique. The viral titer was shown to increase from passage 1 through passage 3, with TCID_50_/ml of 10^3^, 10^4.7^, and 10^6^ for passage 1, 2, and 3, respectively.

### 3.3. Molecular Confirmation of IBDV LC–75 Adapted on Cell Culture

**Figure 2:**
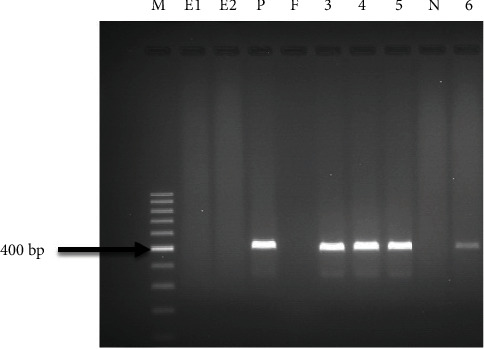
Agarose gel electrophoresis of PCR amplified products of IBDV LC–75 VP2 gene fragments (400 bp). M is the DNA ladder (100 bp, Fermentas), E_1_ and E_2_ is the RNase-free water (extraction control), P is the positive control (IBD vaccine seed D78 strain), and F is the free wells. 400 bp gene fragment of IBDV LC–75 adapted on DF-1 cells: passage 1 (Lane 3), passage 2 (Lane 4), and passage 3 (Lane 5); N is the negative control and Lane 6 is the gene fragment (400 bp) of IBDV LC–75 propagated on CEF cells.

To confirm for IBDV LC–75 on infected-cell cultures (DF-1 cell and CEF cells), a 400 bp of the HVR of IBDV VP2 gene was amplified using a one-step RT-PCR. The PCR products were visualized on agarose gel electrophoresis as 400 bp band specific for VP2 (Figure 2). Bands generated from virus-infected DF-1 and CEF cell homogenates were compared with that of the positive control (IBDV vaccine seed D78 strain), thus confirming the identity of the virus as IBDV.

### 3.4. Safety of DF-1 Cell and CEF Cell-Based IBDV Vaccines

A 10 times higher than the regular dose of each of the study, vaccine was administered to two groups of 15 chickens, followed by a 21 days of postvaccination monitoring. As a result, none of the experimental chickens showed abnormality and death related to vaccination.

### 3.5. Immunogenicity of DF-1 Cell and CEF Cell-Based IBDV Vaccines

**Figure 3:**
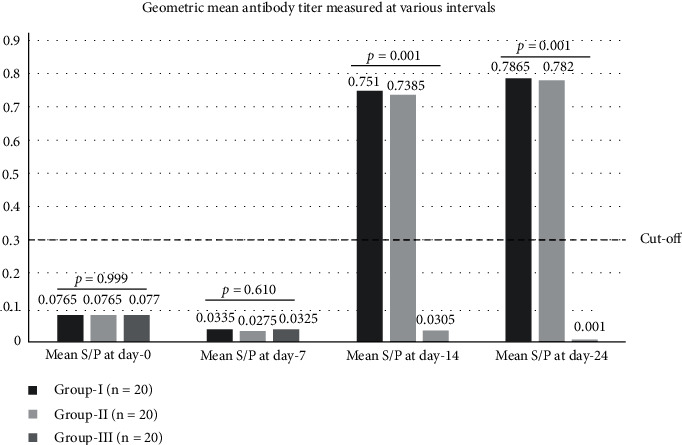
Geometric mean antibody titer measured on prevaccination and postvaccination sera. Group-I chickens (*n* = 20) were vaccinated with DF-1 cell-adapted IBDV vaccine, Group-II chickens (*n* = 20) were vaccinated with CEF cell-based IBDV vaccine, while Group-III served as unvaccinated control. Serum was collected at various intervals: prevaccination, and 7, 14, and 24 days-postvaccination, and was analysed using iELISA. OD values were measured at 450 nm. One-way ANOVA *p* values are indicated to show comparison between the 3 groups. *S*/*P* is the sample to positive ratio.

Geometric mean of serum antibody titer was computed for chickens of both vaccinated (Group-I and Group-II) and unvaccinated groups (Group-III), before vaccination, and 7, 14, and 24 dpv using iELISA. The mean prevaccination *S*/*P* values for Groups-I, -II, and -III were 0.0765 (95% CI: 0.0608-0.0922), 0.0765 (95% CI: 0.0554-0.0976), and 0.0770 (95% CI: 0.0609-0.0931), respectively. The mean *S*/*P* values measured on 7 dpv were 0.0335 (95% CI: 0.0242-0.0428) (Group-I), 0.0275 (95% CI: 0.0191-0.0359) (Group-II), and 0.0325 (95% CI: 0.0218-0.0432) (Group-III). The *S*/*P* values at day 0 and day 7 (*S*/*P* < 0.3) indicated no apparent IBDV specific antibody production in all chickens. In addition, there were no statistically significant differences in mean antibody titers among the different groups at day 0 (*p* = 0.99) and 7 dpv (*p* = 0.61) (Figure 3). However, mean antibody titer increased from 0.0765 (95% CI: 0.0608-0.0922) (baseline) to 0.7510 (95% CI: 0.5499-0.9521) (14 dpv, fold change = 9.8) in Group-I, and from 0.0765 (95% CI: 0.0554-0.0976) (baseline) to 0.7385 (95% CI: 0.5448-0.9322) (14 dpv, fold change = 9.65) in Group-II. The mean antibody titer of unvaccinated chickens remained at 0.0305 at 14 dpv. Slight increment in antibody titer was detected at day 24 postvaccination as compared to the values at 14 dpv. Mean antibody titer of chickens in Group-I slightly raised to 0.7865 (95% CI: 0.6668-0.9062) (fold change = 1.05). Similarly, mean antibody titer of chickens in Group-II subtly increased to 0.7820 (95% CI: 0.6112-0.9528) (fold change = 1.06). Contrarily, Group-III did not show a significant change at 24 dpv. Comparison of mean antibody titers at 14 and 24 dpv showed a statistically significant difference among groups (*p* = 0.001) (Figure 3). Statistical differences between two groups (DF-1 cell-based IBDV vaccine vs. unvaccinated control; CEF-based IBDV vaccine vs. unvaccinated control; DF-1 cell-based IBDV vaccine vs. CEF-based IBDV vaccine) were tested using Tukey's HSD. Chickens that received the DF-1 cell and CEF cell-based IBDV vaccines had a significant difference from unvaccinated groups. However, antibody titer did not show significant difference among the two vaccinated groups (Table 1).

### 3.6. Efficacy of DF-1 Cell and CEF Cell-Based IBDV Vaccines

**Figure 4:**
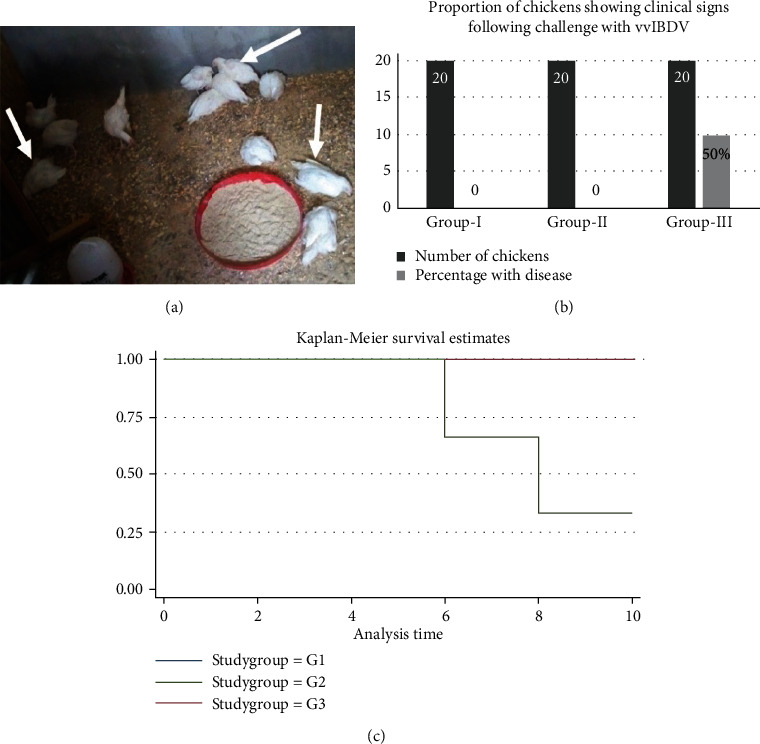
Efficacy of IBDV vaccines against vvIBDV (JF826453). Vaccinated and unvaccinated groups of chicken were challenged with locally isolated vvIBDV (JF826453) with a titer of 10^5.6^ TCID_50_/ml through ocular route. Morbid and dead experimental chickens (a) during the 10 days of follow-up, 50% of the unvaccinated chickens were sick (b) while 30% died (10% at day 6 and 20% at day 8). On the other hand, vaccinated chickens (DF-1 and CEF cell-based vaccines) were completely protected (*p* = 0.005). (c) G1: DF-1 cell-based vaccine, G2: CEF cell-based vaccines, and G3: unvaccinated controls.

Vaccine efficacy was assessed by challenging chickens of vaccinated and unvaccinated groups with locally isolate vvIBDV (JF826453) with a titer of 10^5.6^ TCID_50_/ml intraocularly followed by 10 days of postchallenge monitoring. All chickens that received the DF-1 cell and CEF-based vaccines were fully protected against any clinical signs or death. Conversely, 50% of chickens in the unvaccinated group exhibited depression, ruffled feathers, anorexia, and whitish watery diarrhea starting from 4 days postchallenge (dpc) (Figures 4(a) and 4(b)). Moreover, 30% (10% at day-6 & 20% at day-8) of chickens in this group died by 8 dpc (Figures 4(a) and 4(c)). Log-rank test between vaccinated (Group-I or Group-II) and unvaccinated chickens showed a statistically significant difference (*χ*2 = 7.87, df = 1, *p* = 0.005).

## 4. Discussion

Vaccination is a key tool to prevent IBD in the poultry industry [21]. Currently, the NVI (Bishoftu, Ethiopia) produces intermediate plus IBDV vaccine based on the LC–75 strain grown on CEF. This method of production is otherwise expensive, tedious, and technically inconvenient as it requires continuous preparation of primary CEF cells from SPF eggs, which have to be imported from abroad. Continuous cell lines are robust, easy to work with, and convenient to maintain than primary cells (Rodrigues et al. [38]).

In this study, IBDV LC–75 was propagated on DF-1 cell line, and examined for its safety, immunogenicity, and efficacy (against vvIBDV, JF826453), as an effective substitute to the CEF-based vaccine. CPEs started to appear 7 d.p.i and 6 d.p.i during the 1^st^ and 2^nd^ passage, respectively. Prominent CPEs such as cytoplasmic granulation, cell rounding, aggregation, detachment, and floating were observed 5 d.p.i of passage 3. The CPEs observed on DF-1 and CEF cells in this study, were consistent with CPEs reported when IBDV grew on Vero cells [32], and DF-1 and CEF cells [31, 39] Confirmation of virus identity in CPE positive cultures was accomplished using one-step RT-PCR by amplifying a 400 bp segment of the HVR of the VP2 gene. The one-step RT-PCR offers several advantages. It enables reverse transcription of IBDV RNA into cDNA and then amplification by PCR under a single set of conditions. As it avoids the need to open the reaction tube after initial reverse transcription, the possibility of contamination and the hands-on work required is reduced. Comparably, Barlič-Maganja et al. [40] have amplified 479 bp fragment of the HVR of IBDV VP2. While others have amplified different size amplicons, such as 645 bp [28, 41] and 604 bp [42], which might depend on availability of primers in our study, increasing viral titers of 10^3^ TCID_50_/ml, 10^4.7^ TCID_50_/ml, and 10^6^ TCID_50_/ml were recorded at passage 1, 2, and 3, respectively, showing successful adaptation of the virus on DF-1 cell line. Similar finding has been reported in Vero cell propagated IBDV LC–75 [32]. Consistently, Wang et al. [39] reported that DF-1 cells yielded higher IBD viral titers, which might be due to a stronger affinity of IBDV receptor on DF-1 cells than in CEF cells. However, all IBDV strains are not capable of growing in cell cultures [43], thus DF-1 cells could not be suitable for all IBDV vaccine strains.

The Code of American Federal Regulation recommends a minimum IBD vaccine titer of not less than log_10_^3.40^ TCID_50_/dose for protecting chickens against the disease [44]. In line with this, sufficient viral titers are recorded in this study starting from the 2^nd^ passage which can be used as a vaccine against IBD.

The safety of DF-1 and CEF cell derived IBDV vaccines was assessed by administering chickens a higher than the regular dose (10×) of each vaccine intraocularly. None of the vaccinated chickens showed local or systemic signs. In agreement with this, several authors have reported that intermediate IBDV vaccines are safe and induced less to none immunosuppression of vaccinated chickens [45–47]. However, we did not analyse the gross and microscopic appearance of BF following vaccination, which should be considered in further studies.

After successful adaptation of IBDV LC–75 on DF-1 cells, 0.2 ml (10^6^ TCID_50_/ml) of the virus and 0.2 ml (10^7^ TCID_50_/ml) of CEF-based IBDV vaccine was administered intraocularly to comparatively test for immunogenicity 7, 14, and 24 dpv. As a result, vaccination did not induce detectable serum antibodies 7 dpv as measured by iELISA. In addition, there were no significant differences in mean antibody titers among the different groups (including the unvaccinated control) at day 0 and 7 dpv (*p* > 0.05) suggesting that adaptive immunity would not be produced during the first week postvaccination. Similarly, Kebede et al. [32] did not detect sufficient antibody titer that could be considered positive 7 dpv using Vero cell-adapted IBDV LC–75. Nevertheless, Rasool and Hussain [48] and Amer et al. [49] reported an increased serum antibody titer starting from 7 dpv with live IBDV vaccine. Dissimilarities in IBDV strain, degree of attenuation, viral doses, and chicken immunogenetics may have contributed to the differences observed. However, 85% and 100% of vaccinated chickens in both groups seroconverted 14 and 24 dpv with sufficient antibody titer (*S*/*P* > 0.3), respectively. This agrees with the typical duration of time required for an adaptive immunity to develop following or infection or vaccination [50]. Differences between mean antibody titers between vaccinated and unvaccinated chickens were significant 14 and 24 dpv (*p* < 0.05), while titers were indifferent among vaccinated groups in all measurements (*p* > 0.05) suggesting similar immunogenicity of both vaccines. Moreover, titers significantly varied between 14 and 24 dpv in both vaccinated groups. The clinical significance of such increments can be area of further investigation. As mentioned above, chickens in this study were immunized via the ocular route which resulted in significant serum antibody titer. This aligns with the well-established feature of mucosal vaccines that are known to be good inducers of systemic immunity [51]. The Harderian gland, located behind the eyes of chickens, is the main immune organ suited to mount an effective immune response against ocular vaccination or infection [52].

The protective efficacy of the vaccines in this study was assessed in infection-challenge experiments against vvIBDV (JF826453). Vaccination with both DF-1 and CEF-based IBDV vaccines rendered a complete protection against intraocular challenge with 10^5.6^ TCID_50_/ml vvIBDV 4 weeks postvaccination (JF826453). Again, indicating comparability of the two vaccines in terms efficacy. Likewise, Vero cell-adapted IBDV LC–75 vaccine was able to provide complete protection against vvIBDV [32], corroborating the effectiveness of IBDV LC–75 based vaccines. Contrarily, 50% of unvaccinated chickens in our study suffered signs of depression, ruffled feathers, anorexia, and whitish watery diarrhea in response to ocular challenge 4 dpc. The clinical signs observed were consistent with symptoms of IBD in chickens described elsewhere [53]. Additionally, 30% of chickens in this group died as result of challenge (10% at day 6 and 20% at day 8). Concordantly, Kebede et al. [32] reported 60% of morbidity and 25% of mortality in unvaccinated chickens challenged with vvIBDV. The slight variation in the figures could be due to differences in the strain and dose of the virus, and the breed of chickens used in the experiments.

## 5. Conclusion

In conclusion, the results of this study demonstrated that the DF-1 cell-based preparation of IBDV LC–75 vaccine is comparably immunogenic and efficacious to the CEF-based vaccine. Therefore, DF-1 cell-based IBDV vaccine production is an affordable and convenient method that can replace the complicated primary CEF cell-based approach currently employed at the NVI (Bishoftu, Ethiopia). However, the suitability of DF-1 cell line to support the growth of other IBDV strains and the effect of this vaccine on BF should further be investigated.

## Figures and Tables

**Table 1:** Tukey's HSD between each study group.

	Blood draw			
	Day 0	Day 7	Day 14	Day 24
HSD	0.024	0.024	0.262	0.195
Mean *S*/*P* (GI - GIII)	-0.0005	0.001	0.7205^∗^	0.7855^∗^
Mean *S*/*P* (GII - GIII)	-0.0005	-0.005	0.708^∗^	0.781^∗^
Mean *S*/*P* (GI - GII)	0	0.006	0.0125	0.0045

HSD: honestly significant difference; GI: Group-I, GII: Group-II, and GIII: Group-III unvaccinated control. ^∗^: values greater than HSD values indicating honestly significant difference between two groups. Note: HSD was calculated using the formula provided in the data analysis section.

## Data Availability

The data that support the findings of this study are available from the corresponding author upon request.
